# Effects of improved complementary feeding and improved water, sanitation and hygiene on early child development among HIV-exposed children: substudy of a cluster randomised trial in rural Zimbabwe

**DOI:** 10.1136/bmjgh-2019-001718

**Published:** 2020-01-13

**Authors:** Jaya Chandna, Robert Ntozini, Ceri Evans, Gwendoline Kandawasvika, Bernard Chasekwa, Florence D Majo, Kuda Mutasa, Naume V Tavengwa, Batsirai Mutasa, Mduduzi NN Mbuya, Lawrence H Moulton, Jean H Humphrey, Andrew J Prendergast, Melissa Gladstone, The SHINE Trial Team

**Affiliations:** 1 Department of Women and Child Health, University of Liverpool, Liverpool, UK; 2 Zvitambo Institute for Maternal and Child Health research, Harare, Zimbabwe; 3 Centre for Genomics and Child Health, Blizard Institute, Queen Mary University of London, London, UK; 4 University of Zimbabwe College of Health Sciences, Harare, Zimbabwe; 5 Global Alliance for Improved Nutrition, Washington, DC, USA; 6 Department of International Health, Johns Hopkins Bloomberg School of Public Health, Baltimore, Maryland, USA

**Keywords:** early child development, complementary feeding, sanitation, hand washing, safe drinking water, HIV, HIV-exposed uninfected

## Abstract

**Introduction:**

HIV-exposed uninfected children may be at risk of poor neurodevelopment. We aimed to test the impact of improved infant and young child feeding (IYCF) and improved water, sanitation and hygiene (WASH) on early child development (ECD) outcomes.

**Methods:**

Sanitation Hygiene Infant Nutrition Efficacy was a cluster randomised 2×2 factorial trial in rural Zimbabwe ClinicalTrials.gov NCT01824940). Pregnant women were eligible if they lived in study clusters allocated to standard-of-care (SOC; 52 clusters); IYCF (20 g small-quantity lipid-based nutrient supplement/day from 6 to 18 months, complementary feeding counselling; 53 clusters); WASH (pit latrine, 2 hand-washing stations, liquid soap, chlorine, play space, hygiene counselling; 53 clusters) or IYCF +WASH (53 clusters). Participants and fieldworkers were not blinded. ECD was assessed at 24 months using the Malawi Developmental Assessment Tool (MDAT; assessing motor, cognitive, language and social skills); MacArthur Bates Communication Development Inventories (assessing vocabulary and grammar); A-not-B test (assessing object permanence) and a self-control task. Intention-to-treat analyses were stratified by maternal HIV status.

**Results:**

Compared with SOC, children randomised to combined IYCF +WASH had higher total MDAT scores (mean difference +4.6; 95% CI 1.9 to 7.2) and MacArthur Bates vocabulary scores (+8.5 words; 95% CI 3.7 to 13.3), but there was no evidence of effects from IYCF or WASH alone. There was no evidence that that any intervention impacted object permanence or self-control.

**Conclusions:**

Combining IYCF and WASH interventions significantly improved motor, language and cognitive development in HIV-exposed children.

**Trial registration number:**

NCT01824940.

**Key questions**

**What is already known?**

- Globally, an estimated 43% of children fail to reach their full developmental potential.
- The population of HIV-exposed uninfected (HEU) children is expanding, and reached nearly 15 million in 2017.
- Children who are HEU may be at greater risk of poor early child development than HIV-unexposed children.

**What are the new findings?**

- Compared with standard-of-care, children randomised to combined infant and young child feeding (IYCF) plus water, sanitation and hygiene (WASH) had higher total child development scores as measured by the Malawi Developmental Assessment Tool (mean difference +4.6; 95% CI 1.9 to 7.2).
- Compared with standard-of-care, children randomised to combined IYCF+WASH had higher MacArthur Bates vocabulary scores (+8.5 words; 95% CI 3.7 to 13.3).
- There was no evidence that IYCF or WASH alone affected child development.

**What do the new findings imply?**

- HEU children may be particularly responsive to a package of public health interventions, which may support a targeted intervention approach to ensure that HEU children survive, thrive and reach their full potential.

## Introduction

Globally, 1.4 million HIV-infected women become pregnant each year, predominantly in sub-Saharan Africa. Due to increased coverage of prevention of mother-to-child transmission (PMTCT) interventions, the number of HIV-exposed uninfected (HEU) children is expanding, and reached nearly 15 million in 2017.^1^ HEU children have higher mortality and more frequent and more severe infections, anaemia and growth faltering than children born to HIV-negative mothers (HIV-unexposed children).^2^ Since stunting (linear growth faltering),^3^ anaemia^4^ and inflammation^5^ are all associated with impaired neurodevelopment, HEU children may be at greater risk of poor early child development (ECD) than HIV-unexposed children, although empirical evidence is limited.^6^ Together these observations suggest that interventions to reduce stunting, anaemia and infections may have particular benefits for the growing population of HEU children, including enhanced neurodevelopment.

The Sanitation Hygiene Infant Nutrition Efficacy (SHINE) trial was designed to assess the individual and combined effects of an infant and young child feeding (IYCF) intervention and a household water, sanitation and hygiene (WASH) intervention on stunting and anaemia in HIV-unexposed and HIV-exposed Zimbabwean children.^7^ The WASH intervention was designed to reduce exposure to faecal microbes, and thereby prevent a subclinical inflammatory disorder of the gut termed environmental enteric dysfunction (EED), which may mediate stunting, anaemia and reduced ECD. We previously reported that the IYCF intervention reduced stunting and anaemia in HIV-unexposed^8^ and HIV-exposed^9^ children at 18 months of age, but the WASH intervention had no impact on either of these trial outcomes. A substudy, assessing the effects of the randomised interventions on ECD at 24 months of age, showed there was overall little impact of either intervention on neurodevelopment among HIV-unexposed children.^10^ Here, we report the impact of the randomised interventions on ECD at 24 months among HIV-exposed children, in whom we hypothesised the trial interventions may have distinct effects compared with HIV-unexposed children.

## Methods

### ​Study design and randomisation

The SHINE trial design has been reported previously; the protocol and statistical analysis plan are available at https://osf.io/w93hy Briefly, SHINE was a cluster randomised, community-based 2×2 factorial trial conducted in two contiguous rural districts in Zimbabwe. Clusters were defined as the catchment area of 1–4 village health workers (VHWs) employed by the Ministry of Health and Child Care, and were allocated to one of four treatment groups (standard-of-care (SOC), IYCF, WASH, IYCF +WASH) at a public randomisation event. A highly constrained randomisation technique achieved balance across arms for 14 variables related to geography, demography, water access and sanitation coverage. Between 22 November 2012 and 27 March 2015, VHWs identified pregnant women and referred them to trial research nurses, who enrolled women permanently residing in the study area into SHINE following written informed consent. HIV prevalence among antenatal women in the study area was 15%; we prespecified that analysis of all outcomes would be stratified by maternal HIV status.

### Trial interventions

Interventions were informed by extensive formative research and piloting.^7^ Behavioural change modules using interactive tools to deliver specific messages were provided by arm-specific VHWs; lesson plans and intervention tools are publicly accessible at https://osf.io/w93hy. All women were scheduled to receive 15 VHW visits between enrolment and 12 months postpartum; other family members were encouraged to participate. At each visit, previous information was reviewed before introducing new information to create a sequenced, integrated, longitudinal intervention. Between 13 and 17 months, VHWs visited monthly, providing routine care and, in active arms, delivering intervention supplies; during these visits VHWs informally encouraged participants to practise relevant behaviours. At 18 months, a review module was delivered in all arms. Key messages and supplies are outlined below, with more detail provided in the online supplementary methods:

10.1136/bmjgh-2019-001718.supp1 Supplementary data

- SOC Promotion of exclusive breast feeding to 6 months, uptake of antenatal and neonatal care, PMTCT, immunisations, family planning.
- IYCF All SOC messages plus (1) importance of nutrition for infant health, growth and development; (2) feeding nutrient-dense food and 20 g small-quantity lipid-based nutrient supplement (SQ-LNS; Nutriset, Malaunay, France) daily from 6 to 18 months; (3) processing locally available foods to facilitate mastication and swallowing; (4) feeding during illness; (5) dietary diversity. Monthly delivery of SQ-LNS from 6 to 18 months.
- WASH All SOC messages plus (1) safe disposal of faeces; (2) hand-washing with soap at key times; (3) protection of infants from geophagia and animal faeces ingestion; (4) chlorination of drinking water and (5) hygienic preparation of complementary food. Ventilated improved pit latrine constructed within 6 weeks of enrolment; two hand-washing stations; plastic mat and play yard; monthly delivery of soap and chlorine (WaterGuard, Nelspot, Zimbabwe).
- IYCF±WASH All SOC, IYCF and WASH interventions.

A latrine was constructed in non-WASH arms following trial completion. Masking for participants and fieldworkers was not possible, but investigators were blinded to trial arm.

### ​Maternal HIV testing

Local clinics undertook antenatal HIV testing and provided antiretroviral therapy (ART) to HIV-positive women. PMTCT guidelines in Zimbabwe changed from WHO Option B (combination ART for all HIV-positive women during the pregnancy and breast feeding) to Option B+ (lifelong ART for pregnant and breastfeeding women) during the trial (from November 2013). In addition to local clinic services, we offered home HIV testing to mothers at baseline using an anti-HIV antibody rapid test algorithm (Alere Determine HIV-1/2 test, confirmed by INSTI HIV-1/2 test if positive); testing was repeated at 32 gestational weeks to detect HIV seroconversion during pregnancy. Women testing HIV-positive had CD4 counts measured (Alere Pima, Abbott) and were referred to local clinics for ART. The trial did not measure HIV viral loads.

### ​Child HIV testing

HIV-positive women were encouraged to attend local clinics at 4–6 weeks post partum for early infant diagnosis, initiation of cotrimoxazole prophylaxis and, for HIV-infected children, ART initiation. Infants born to HIV-positive mothers were eligible for enrolment into a substudy in which biological specimens were collected longitudinally. For substudy infants, blood was tested for HIV (by PCR or rapid test, depending on age and sample) at 1, 3, 6, 12 and 18 months; infants not enrolled into the substudy were tested at 18 months. HEU children were defined as being born to HIV-positive mothers and testing HIV-negative at 18 months of age. HIV-exposed children, who were not tested at 18 months because of maternal refusal, missed visits or failure to obtain a specimen or children who had inconclusive/discordant HIV results after retesting, were classified as HIV unknown. All HIV-positive children were referred to clinics for ART initiation.

### ​Data collection

Research nurses made home visits at baseline (~2 weeks after consent), 32 weeks’ gestation and at 1, 3, 6, 12 and 18 months postpartum to assess maternal and household characteristics and trial outcomes. Intervention uptake was assessed by participant behaviours at the 12-month postnatal visit.

### ECD substudy

Infants who completed the trial and turned 2 years of age (allowable range 102–112 weeks) between 1 March 2016 and 30 April 2017 were eligible to join the ECD substudy. Children were enrolled either during the 18-month trial endpoint visit, or following the 18 month SHINE visit but before the child turned 2 years of age.

### ​Assessment tools

A team of 11 research nurses completed 3 weeks of residential training in ECD assessment by the team psychologist (JC) and a neurodevelopmental paediatrician (MG). Several domains of ECD were assessed.

1. Malawi Developmental Assessment Tool (MDAT), measuring child development in four domains: gross and fine motor coordination, language and social. Fine motor, language and social domains also measure components of cognitive development.^11^ The MDAT was initially validated in Malawi (a very similar setting to Zimbabwe) and then piloted on 50 Zimbabwean children.
2. MacArthur Bates Communicative Development Inventories (CDI), assessing language according to maternal report using vocabulary and grammar checklists.^12^ The test was specifically adapted for Shona speakers using a detailed protocol approved by the CDI team.^13 14^
3. A-not-B test, assessing object permanence and cognition.^15^
4. Self-control task, assessing impulsivity.^16^

### ​Study outcomes

The ECD substudy design and outcomes were prespecified in the study protocol and statistical analysis plan (https://osf.io/w93hy). The primary outcomes were MDAT total (out of 138), gross motor (out of 36), fine motor (out of 36), social (out of 30) and language (out of 36) scores; MacArthur Bates CDI vocabulary checklist (number of words known out of 99); A-not-B score (out of 10); and the proportion of children with self-control. The secondary outcomes were the proportion of children using the progressive tense, using plurals or combining two words (MacArthur Bates CDI grammar checklist).

Children with severe motor, visual, hearing or learning impairments as determined by the Washington Group questionnaire (child version)^17^ were excluded from analyses and referred to local clinics.

### ​Validation and quality control

Every 6 months, nurses underwent refresher training and standardisation. Each nurse was observed while conducting an ECD assessment while a gold-standard assessor double-scored the assessment; percentage agreement had to be >85% for certification, with retraining and retesting required for those who did not meet this threshold. In addition, all 11 nurses concurrently scored the same child: average interclass correlations across standardisations were: MDAT 0.88 (95% CI 0.82 to 0.94); MacArthur Bates 0.94 (95% CI 0.90 to 0.96); A-not-B 0.85 (95% CI 0.80 to 0.90) and self-control task 0.80 (95% CI 0.76 to 0.85). Supportive supervision was undertaken during monthly field visits and nurses were provided with corrective or reinforcing feedback. A 5% subsample of assessments were video recorded, reviewed and double scored by a psychologist (JC) and a neurodevelopmental paediatrician with Shona language proficiency (GK). Percentage agreement for these video-taped assessments was 93% for MDAT fine motor, 90% for MDAT language, 97% for A-not-B and 91% for the self-control task.

### ​Statistical analysis

All analyses were intention to treat at the child level. The absolute difference in mean score between treatment groups was estimated for tests with continuous outcomes. For tests with dichotomous outcomes, the relative risk (RR) of passing was compared between treatment groups. Primary analyses used generalised estimating equations to account for within-cluster correlation, containing two dummy variables for the two interventions, representing the main effect of the IYCF intervention (the two IYCF-containing groups compared with the two groups without IYCF) and the WASH intervention (the two WASH-containing groups compared with the two groups without WASH), unadjusted for other covariates, with an exchangeable working correlation structure. For each outcome, we estimated the statistical interaction between the IYCF and WASH interventions. When the interaction was significant (p<0.05 according to the Wald test), results are based on a regression model with three dummy variables to represent IYCF, WASH and IYCF +WASH compared with SOC, instead of the model with two terms. Adjusted analyses controlled for prespecified baseline covariates (mother’s mid-upper arm circumference, mother’s education, mother’s employment, maternal health perception, maternal capabilities, improved latrine, low birth weight, prematurity, sex, calendar month, fieldworker, decimal age), which were initially assessed in bivariate analyses to identify those with an important association with the outcome (for dichotomous outcomes: p<0.2 or RR >2.0 or <0.5; for continuous outcomes: p<0.2 or difference >0.25 SD). Selected covariates were entered in a multivariable regression model; a forward stepwise selection procedure was implemented with p<0.2 to enter. A log-binomial specification was used to estimate RRs. Methods for comparing study arms while handling within-cluster correlation included multinomial and ordinal regression models with robust variance estimation, and Somers’ D for medians, were all implemented in Stata V.14.

In a sensitivity analysis, HIV-positive and HIV-unknown children were excluded. A subgroup analysis by child gender was planned if there was a significant interaction between gender and study arms (as defined above).

### ​Sample size

The sample size of the ECD substudy was based on detecting clinically relevant differences among HIV-unexposed children^10^; there was no specific sample size calculation for HIV-exposed children.

### ​Patient and public involvement

We did not directly include patient and public involvement in this trial, but all community activities were discussed with traditional and elected leaders in both study districts, who provided advice through the District Health Executive, Social Services Committee and Rural District Council. A film of the SHINE trial is being made with community participation to capture the experience of being involved in a community-wide trial. The film will be screened in the two rural districts where SHINE was conducted.

### ​Role of funder

Study funders approved the trial design, but were not involved in data collection, analysis or interpretation, nor decisions related to publication. The corresponding author had full access to all study data and ultimate responsibility for the decision to submit for publication.

### ​Trial oversight and registration

An independent data safety and monitoring board reviewed interim adverse event data.

## Results

**Figure 1:**
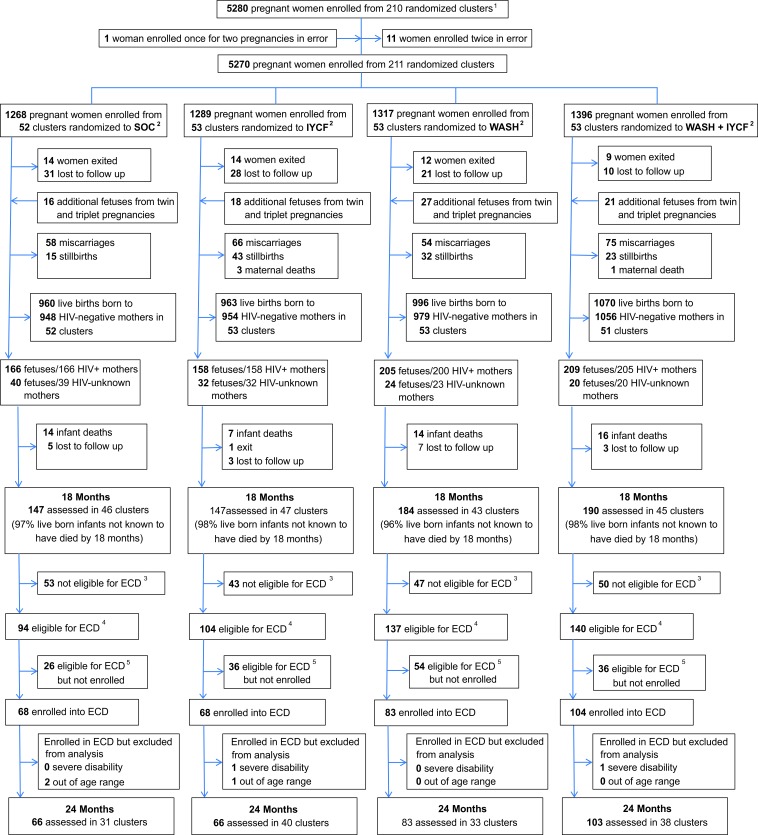
Flow of participants through the trial. 1A total of 212 clusters were randomised, 53 in each of the four trial arms. After randomisation, one cluster was excluded as it was determined to be in an urban area, one cluster was excluded as the VHW covering it mainly had clients outside the study area, and one more was merged into a neighbouring cluster based on subsequent data on VHW coverage. Three new cluster designations were created due to anomalies in the original mapping: for two of these, the trial arm was clear; the third contained areas that were in two trial arms and was assigned to the underrepresented arm, resulting in 53 clusters in each arm. All of this occurred before enrolment began. When enrolment was completed, however, there was one cluster (SOC) in which no women were enrolled, leaving a total of 211 clusters available for analysis. 2IYCF, infant and young child feeding; SOC, standard- of-care; WASH, water, sanitation and hygiene. 3Children were not eligible for the early child development (ECD) substudy if they turned 2 years of age (allowable range 102–112 weeks) before 1 March 2016. 4Children were eligible for the ECD substudy if they turned 2 years of age (allowable range 102–112 weeks) between 1 March 2016 and 30 April 2017. 5A total of 152 children were eligible but not enrolled: 94 (62%) had relocated temporarily or permanently from their study home; 41 (27%) were not reachable by telephone or home visit to determine availability and interest in joining the ECD substudy; 8 (5%) declined; 4 (3%) could not be scheduled at a mutually agreeable time within the required age window; and 3 (2%) died between 18 and 24 months of age. VHW, village health worker.

Among 5280 enrolled pregnant women, 726 tested HIV-positive during the pregnancy; of 738 infants born to these mothers, 475 were eligible for the ECD substudy, and 323 (68% of eligible) were enrolled from 142 clusters (figure 1) during the period of enrolment (between 1 March 2016 and 30 April 2017). Of the 152 children not enrolled, 94 (62%) had relocated temporarily or permanently from their study home; 41 (27%) were not reachable by telephone or home visit to determine availability and interest in joining the ECD substudy; 8 (5%) declined; 4 (3%) could not be scheduled at a mutually agreeable time within the required age window; and 3 (2%) died between 18 and 24 months of age.

### ​Baseline characteristics

At baseline, approximately half of all household members practised open defecation, and just over one-third had a household latrine (table 1). About 40% of households consumed a minimally diverse diet. Among mothers, mean (SD) CD4 count was 461 (218) cells/µL; 88% and 67% received ART and cotrimoxazole, respectively, during the pregnancy. There were some minor baseline imbalances in improved latrine ownership, observed chicken faeces in households, wealth index, mode of delivery and birth weight between arms. HIV-positive mothers who enrolled in the ECD substudy were about 3 years older than those who did not enrol, SOC and IYCF +WASH enrolled mothers had higher institutional delivery (92% vs 74%) while IYCF and WASH arms were similar; other baseline characteristics of mother–infant dyads who enrolled or did not enrol were similar (online supplementary table 1).

10.1136/bmjgh-2019-001718.supp2 Supplementary data

**Table 1:** Maternal, household and infant baseline characteristics of HIV-positive mothers and their liveborn infants in the early child development substudy

Baseline characteristics*	Standard-of-care	IYCF	WASH	IYCF+WASH
Mothers	68	67	81	102
Infants	68	68	83	104
Mothers completing baseline visit	67	65	80	101
Household characteristics				
Median no of occupants (IQR)	5 (3–6)	4 (3–6)	5 (3–6)	4 (3–6)
Wealth quintile:†				
1 (lowest)	16/68 (23.5%)	14/67 (20.9%)	20/81 (24.7%)	28/102 (27.5%)
2	16/68 (23.5%)	13/67 (19.4%)	15/81 (18.5%)	19/102 (18.6%)
3	12/68 (17.7%)	13/67 (19.4%)	19/81 (23.5%)	17/102 (16.7%)
4	8/68 (11.8%)	17/67 (25.4%)	11/81 (13.6%)	17/102 (16.7%)
5 (highest)	14/68 (20.6%)	8/67 (11.9%)	13/81 (16.1%)	19/102 (18.6%)
Electricity				
Connected to power grid	3/66 (4.6%)	3/65 (4.6%)	0/78 (0.0%)	3/100 (4.0%)
Other power source				
Use a generator	2/66 (3.0%)	3/65 (4.6%)	0/78 (0.0%)	3/100 (3.0%)
Use solar power	45/66 (68.2%)	44/65 (67.7%)	56/78 (71.8%)	64/100 (64.0%)
No electricity	19/66 (28.8%)	18/65 (27.7%)	22/78 (28.2%)	33/100 (33.0%)
Sanitation				
Household members defecate in the open	177/302 (58.6%)	151/282 (53.6%)	182/337 (54.0%)	196/426 (46.0%)
Any latrine at household	18/66 (27.3%)	28/62 (45.2%)	29/77 (37.7%)	37/96 (38.5%)
Improved latrine at household	17/66 (25.8%)	24/62 (38.7%)	25/77 (32.5%)	36/96 (37.5%)
Improved latrine with well-trodden path and not shared with other households and not used for storage	12/64 (18.8%)	28/60 (31.7%)	29/76 (27.6%)	23/94 (24.5%)
Water				
Main source of household drinking water is improved	41/66 (62.1%)	36/62 (58.1%)	44/77 (57.1%)	59/96 (61.5%)
Treat drinking water to make it safer	13/64 (20.3%)	11/62 (17.7%)	10/77 (13.0%)	13/96 (13.5%)
Median one-way walk time to fetch water (IQR); min	10 (5–20)	9 (3–15)	10 (5–15)	9 (5–20)
Mean water volume collected per person in past 24 hours (SD); L	8.7 (4.6)	9.4 (6.7)	9.1 (6.7)	10.3 (8.2)
Hygiene				
Hand-washing station at household	3/54 (5.6%)	4/62 (6.5%)	11/74 (14.9%)	15/91 (16.5%)
Hand-washing station with water and rubbing agent	0/54 (0.0%)	0/60 (0.0%)	0/74 (0.0%)	1/91 (1.1%)
Improved floor‡	31/65 (47.7%)	35/65 (53.9%)	35/77 (45.5%)	48/98 (49.0%)
Median no of chickens (IQR)	4 (1–8)	6 (2–10.5)	5 (3–8)	4 (1–8)
Livestock observed inside home	28/66 (42.4%)	19/67 (28.4%)	34/79 (43.0%)	33/99 (33.3%)
Faeces observed in yard	25/65 (38.5%)	18/66 (27.3%)	27/78 (34.6%)	17/98 (17.4%)
Diet quality and food security				
Household meets minimum dietary diversity§	21/55 (38.2%)	22/57 (38.6%)	24/74 (32.9%)	37/83 (44.6%)
Median Coping Strategies Index score¶ (IQR)	4 (0–9.5)	2 (0–15)	3 (0–11)	1 (0–9)
Maternal characteristics				
Mean age (SD), years	30.8 (5.7)	30.9 (7.2)	30.4 (5.7)	30.9 (5.8)
Mean height (SD), cm	161.3 (7.8)	160.3 (6.4)	160.5 (5.3)	159 (6.6)
Mean mid-upper arm circumference (SD), cm	26.8 (3.4)	26.3 (3.6)	26.4 (2.2)	26.5 (2.8)
Positive microscopy for *Schistosoma haematobium*	6/67 (9.0%)	4/65 (6.2%)	12/78 (15.4%)	9/101 (8.9%)
Mean years of completed schooling (SD)	9.5 (1.8)	9.1 (2.1)	8.7 (2.2)	9.3 (2.1)
Median parity (IQR)	3 (1–3)	2 (1–3)	3 (2–4)	2 (1–3)
Married	61/64 (95.3%)	59/63 (93.7%)	71/73 (97.3%)	85/92 (92.4%)
Employed	5/65 (7.7%)	5/65 (7.7%)	8/78 (10.3%)	8/100 (8.0%)
Religion				
Apostolic	34/63 (54.0%)	32/63 (50.8%)	34/74 (46.0%)	46/93 (49.5%)
Other christian religions	25/63 (39.7%)	25/63 (39.7%)	30/74 (40.5%)	41/93 (44.1%)
Other	4/63 (6.4%)	6/63 (9.5%)	10/74 (13.5%)	6/93 (6.5%)
HIV disease severity and treatment				
Mean CD4 count in pregnancy (SD)**, cells/µL	474 (180)	478 (186)	421 (187)	470 (217)
Antiretroviral therapy during pregnancy††	60/68 (88.2%)	60/67 (89.6%)	65/81 (80.3%)	90/102 (88.2%)
Cotrimoxazole prophylaxis during pregnancy‡‡	40/68 (58.8%)	42/67 (62.7%)	55/81 (67.9%)	63/102 (61.8%)
Infant characteristics				
Female	33/68 (48.5%)	32/63 (47.1%)	39/83 (47.0%)	55/104 (52.9%)
Mean birth weight (SD), kg	3.03 (0.45)	2.94 (0.49)	3.07 (0.66)	3.02 (0.50)
Low birth weight <2500 g	8/68 (11.8%)	10/68 (14.7%)	6/83 (7.2%)	11/104 (10.6%)
Institutional delivery	59/64 (92.2%)	55/66 (83.3%)	67/79 (84.8%)	85/92 (92.4%)
Vaginal delivery	62/66 (93.9%)	57/62 (87.7%)	76/80 (95.0%)	90/98 (91.8%)

*Baseline variables presented for mothers who had live births; maternal and household data were collected about 2 weeks after consent (~14 weeks gestation); this gap created opportunity for lost to follow-up between consent and baseline, thus the number of mothers completing the baseline visit is fewer than the number of mothers with live births. Baseline for infants was at birth. Values are %, unless noted. For variables where (n) is not stated, <3% of data are missing based on number of baseline visits completed.

†Chasekwa *et al*.^23^

‡Improved floor defined as concrete, brick, cement or tile. Unimproved floor defined as mud, earth, sand or dung.

§FAO, FHI 360. Minimum Dietary Diversity for Women: A Guide for Measurement. Rome: FAO. 2016.

¶Maxwell *et al*.^24^

**CD4 count at baseline visit, or at 32 gestational week visit if no baseline result.

††Includes any exposure to antiretroviral therapy during pregnancy; Includes anydocumented exposure to anti-retroviral therapy during pregnancy.

‡‡Includes any exposure to co-trimoxazole during pregnancy; Includes any documented exposure to co-trimoxazole during pregnancy.

IYCF, infant and young child feeding; WASH, water, sanitation and hygiene.

### ​Delivery and uptake of interventions

Across randomised arms, the fidelity of providing intervention supplies (WASH hardware, liquid soap, chlorine and SQ-LNS) was high, and >90% of expected behavioural change modules were delivered by VHWs (table 2). Open defecation was virtually eliminated in the WASH arms: <1% households reported practising open defecation compared with half in non-WASH arms. Among WASH compared with non-WASH households, fewer mothers reported ever seeing their child ingest soil and chicken faeces. Over 90% of children in all treatment arms were still breast feeding at 12 months. A higher proportion of infants in the IYCF arms had consumed a diet that met minimum dietary diversity and had consumed animal-source, iron-rich and vitamin A-rich foods in the previous day. Almost all children in the IYCF arms had consumed SQ-LNS in the previous 24 hours.

**Table 2:** Intervention delivery and participant uptake by treatment group

Delivery of hardware, commodities and behavioural change modules*	Data source	SOC†	IYCF†	WASH†	IYCF plus WASH†	Combined WASH arms‡	Combined non-WASH arms‡	P value	Combined IYCF arms§	Combined non-IYCF arms§	P value
Children with 24-month outcomes (on whom inferences are based), n	Trial logs	66	66	83	103	186	132		169	149	
WASH supplies											
SHINE-installed ventilated improved pit latrine	Trial logs	N/A	N/A	83/83 (100.0%)	101/103 (98.1%)	184/186 (98.9%)	N/A	–	N/A	N/A	–
Two hand-washing stations (Tippy Taps) delivered	Trial logs	N/A	N/A	83/83 (100.0%)	103/103 (100.0%)	186/186 (100.0%)	N/A	–	N/A	N/A	–
Baby mat delivered	Trial logs	N/A	N/A	81/83 (97.6%)	103/103 (100.0%)	184/186 (98.9%)	N/A	–	N/A	N/A	–
Play yard delivered	Trial logs	N/A	N/A	80/83 (96.4%)	103/103 (100.0%)	183/186 (98.4%)	N/A	–	N/A	N/A	–
Median liquid soap deliveries (IQR) (max=20)	Trial logs	N/A	N/A	20 (19–20)	20 (20–20)	20 (19–20)	N/A	–	N/A	N/A	–
Received at least 16 (80% of expected) soap deliveries	Trial logs	N/A	N/A	76/83 (91.6%)	98/103 (95.2%)	174/186 (93.6%)	N/A	–	N/A	N/A	–
Water Guard deliveries, median (IQR) (max=15)	Trial logs	N/A	N/A	15 (15–15)	15 (15–15)	15 (15–15)	N/A	–	N/A	N/A	–
Received at least 12 (80% of expected) Water Guard deliveries	Trial logs	N/A	N/A	76/83 (91.6%)	96/103 (93.2%)	172/186 (92.5%)	N/A	–	N/A	N/A	–
IYCF supplies											
Median SQ-LNS deliveries (IQR) (max=13)	Trial logs	N/A	13 (13; 13)	N/A	13 (13; 13)	N/A	N/A	–	13 (13; 13)	N/A	–
Received at least 12 (80% of expected) SQ-LNS deliveries	Trial logs	N/A	58/66 (89.2%)	N/A	95/103 (92.2%)	N/A	N/A	–	153/169 (90.5%)	N/A	–
Behavioural change modules											
Median intervention modules (IQR), (max=15)	VHW report	15 (13–15)	15 (14–15)	15 (14–15)	15 (15–15)	15 (14–15)	15 (14–15)	0.249	15 (15–15)	15 (14–15)	0.016
Completed intervention modules (% due)	VHW report	1105/1239 (89.2%)	1566/1629 (96.1%)	2006/2140 (93.7%)	2985/3080 (96.9%)	4991/5220 (95.6%)	2671/2868 (93.1%)	0.256	4551/4709 (96.6%)	3111/3379 (92.1%)	0.020
**Participant uptake of promoted behaviours at 12-month visit**											
Mothers with 12 and 24 months outcomes	Trial logs	60	60	74	95	169	120		155	134	
Children with 12 and 24 months outcomes	Trial logs	60	61	76	97	173	121		158	136	
WASH behaviours											
Household members who practise open defecation	Maternal report	112/196 (57.1%)	101/231 (43.7%)	2/311 (0.6%)	0/416 (0.0%)	2/727 (0.3%)	213/427 (49.9%)	<0.001	N/A	N/A	–
Any latrine at household	Observation	11/60 (18.3%)	26/60 (43.3%)	75/75 (100.0%)	92/92 (100.0%)	167/167 (100.0%)	37/120 (30.8%)	<0.001	N/A	N/A	–
Improved latrine at household	Observation	11/60 (18.3%)	16/60 (26.7%)	75/75 (100.0%)	92/92 (100.0%)	169/169 (100.0%)	27/120 (22.5%)	<0.001	N/A	N/A	–
Improved latrine at household with well-trodden path, not used for storage, and not shared with other households	Observation and maternal report	9/60 (15.0%)	13/60 (21.7%)	66/75 (88.0%)	78/92 (84.8%)	144/167 (86.2%)	22/120 (18.3%)	<0.001	N/A	N/A	–
Hand-washing station at household	Observation	2/55 (3.6%)	3/59 (5.1%)	75/75 (100.0%)	93/94 (98.9%)	168/169 (99.4%)	5/114 (4.4%)	<0.001	N/A	N/A	–
Hand-washing station with water and rubbing agent at household	Observation	1/54 (1.9%)	0/57 (0.0%)	62/72 (86.1%)	69/82 (84.2%)	131/154 (85.1%)	1/111 (0.9%)	<0.001	N/A	N/A	–
Ever treats drinking water to make it safer	Maternal report	6/60 (10.0%)	12/60 (20.0%)	63/75 (84.0%)	83/93 (89.3%)	145/167 (86.9%)	18/120 (15.0%)	<0.001	N/A	N/A	–
Disposes rinse water from cleaning infant nappies with faeces in a latrine	Maternal report	9/160 (15.0%)	20/59 (33.9%)	58/76 (76.3%)	68/88 (77.3%)	126/164 (76.8%)	29/119 (24.4%)	<0.001	N/A	N/A	–
Play space is visibly clean	Observation	N/A	N/A	66/71 (93.0%)	84/92 (91.3%)	150/163 (92.0%)	N/A	N/A	N/A	N/A	–
Child ever observed to eat soil	Maternal report	40/60 (66.7%)	40/61 (65.6%)	26/73 (35.6%)	19/93 (20.4%)	45/166 (27.1%)	80/121 (66.1%)	<0.001	N/A	N/A	–
Child ever observed to eat chicken faeces	Maternal report	8/60 (13.3%)	8/61 (13.1%)	3/73 (4.1%)	3/92 (3.3%)	6/165 (3.6%)	16/121 (13.2%)	0.005	N/A	N/A	–
IYCF behaviours											
Child is still breast feeding	Maternal report	51/60 (85.0%)	56/61 (91.8%)	69/75 (92.0%)	87/94 (92.6%)	N/A	N/A	–	143/155 (92.3%)	120/135 (88.9%)	0.338
Mother reports correct ways to feed child during and after illness	Maternal report	44/59 (74.6%)	45/61 (73.8%)	52/75 (69.3%)	71/93 (76.3%)	N/A	N/A	–	116/154 (75.3%)	96/134 (71.6%)	0.463
Infant diet met minimum dietary diversity in past 24 hours	Maternal report	35/60 (58.3%)	40/61 (65.6%)	32/71 (45.1%)	61/90 (67.8%)	N/A	N/A	–	101/151 (66.9%)	67/131 (51.2%)	0.010
Infant consumed iron-rich food in the past 24 hours	Maternal report	38/60 (63.3%)	61/61 (100.0%)	33/73 (45.2%)	88/93 (94.6%)	N/A	N/A	–	149/154 (96.8%)	71/133 (53.4%)	<0.001
Infant consumed animal-source food in the past 24 hours	Maternal report	44/60 (73.3%)	47/61 (77.1%)	41/74 (55.4%)	67/91 (73.6%)	N/A	N/A	–	114/152 (75.0%)	85/134 (63.4%)	0.031
Infant consumed vitamin A-rich food in the past 24 hours	Maternal report	36/60 (60.0%)	48/61 (78.7%)	55/75 (73.3%)	75/93 (80.7%)	N/A	N/A	–	123/154 (79.9%)	91/135 (67.4%)	0.018
SQ-LNS consumed in previous 24 hours	Maternal report	N/A	59/61 (96.7%)	N/A	78/91 (85.7%)	N/A	N/A	–	137/152 (90.1%)	N/A	N/A

P values were adjusted for clustering effect; depending on the variable type, XTGEE, multinomial and ordinal logistic regression models with robust variance estimation, and Somers’ D for medians, were used for comparing arms accounting for within-cluster correlation.

*Data are n/N (%), unless otherwise indicated.

†Combined WASH collapses the two WASH-containing arms (WASH and WASH +IYCF); non-WASH collapses the two arms not including WASH (SOC and IYCF).

‡Combined IYCF collapses the two IYCF-containing arms (IYCF and WASH +IYCF); non-IYCF collapses the two arms not including IYCF (SOC and WASH).

IYCF, infant and young child feeding; N/A, not applicable; N/A, Not Applicable; SHINE, Sanitation Hygiene Infant Nutrition Efficacy; SOC, standard-of-care; SQ-LNS, small-quantity lipid-based nutrient supplement; WASH, water, sanitation and hygiene; XTGEE, Generalised Estimating Equation.

### ​Effect of randomised intervention on ECD outcomes

The age at the ECD assessment visit was very similar across trial arms (SOC: mean (SD) 105.4 (2.2) weeks; IYCF: 105.0 (2.0) weeks; WASH: 105.1 (1.9) weeks and IYCF+WASH 105.6 (2.1) weeks). Of the 323 enrolled children, two (1%) were excluded due to severe disability, and three (1%) were excluded because they were subsequently found to be outside the predefined age window (102–112 weeks). Two children (both in the IYCF+WASH arm) were assessed after the substudy enrolment period ended, but were otherwise eligible and were included. The final analysis therefore included 318 HIV-exposed children (figure 1), of whom 6 were HIV-positive, 300 HEU and 12 HIV-unknown.

There was a significant interaction between the WASH and IYCF interventions for the total MDAT score and MacArthur Bates CDI vocabulary checklist; therefore, scores for these tests were analysed and presented separately for the four treatment arms. There was no interaction between the randomised interventions for the object permanence and self-control tests; therefore, the main effects of IYCF and WASH are presented for these tests.

### ​MDAT scores

At 24 months, the total MDAT score was higher among children in the IYCF +WASH group compared with the SOC group (unadjusted difference +4.6; 95% CI 1.9 to 7.2). This difference corresponds to a 0.50 SD increase in total MDAT score, and was driven by higher scores in all components of the MDAT test (table 3). In adjusted analyses, the total MDAT score remained significantly higher in the IYCF +WASH group (adjusted difference +3.1; 95% CI 0.9 to 5.3); differences in individual components of the MDAT score were attenuated and no longer reached statistical significance for the fine motor and language component scores. There was no evidence of effect from the IYCF or WASH interventions alone, either on total MDAT score or any of the MDAT component scores.

**Table 3:** Effect of WASH and IYCF Inventories on early child development at 24 months among HIV-exposed children

Primary continuous outcomes	Effects by each randomised arm compared with the SOC ARM						
	Treatment group	N	Mean (SD)	Unadjusted difference (95% CI)	P value	Adjusted difference* (95% CI)	P value
**MDAT total score**	SOC	66	90.9 (8.2)	0.0 (ref)		0.0 (ref)	
	IYCF	66	91.7 (8.8)	0.81 (−1.99 to 3.61)	0.572	−0.91 (−3.40 to 1.58)	0.476
	WASH	83	89.6 (9.2)	−1.26 (−3.80 to 1.28)	0.330	−1.63 (−4.26 to 0.99)	0.222
	IYCF+WASH	103	95.3 (9.0)	4.57 (1.91 to 7.23)	0.001	3.05 (0.86 to 5.25)	0.006
**MDAT gross motor**	SOC	66	23.1 (2.8)	0.0 (ref)		0.0 (ref)	
	IYCF	66	23.4 (2.7)	0.38 (−0.50 to 1.27)	0.398	0.01 (−0.88 to 0.91)	0.977
	WASH	83	22.7 (3.2)	−0.25 (−1.00 to 0.49)	0.504	−0.57 (−1.39 to 0.25)	0.174
	IYCF+WASH	103	24.3 (3.3)	1.50 (0.53 to 2.47)	0.002	0.84 (0.08 to 1.61)	0.031
**MDAT fine motor**	SOC	66	23.0 (2.4)	0.0 (ref)		0.0 (ref)	
	IYCF	66	22.7 (3.5)	−0.31 (−1.37 to 0.74)	0.558	−0.50 (−1.52 to 0.51)	0.329
	WASH	83	22.9 (2.6)	−0.10 (−0.86 to 0.66)	0.804	−0.21 (−1.11 to 0.68)	0.637
	IYCF+WASH	103	23.8 (2.6)	0.74 (−0.02 to 1.50)	0.055	0.59 (−0.21 to 1.38)	0.148
**MDAT language**	SOC	66	20.7 (3.7)	0.0 (ref)		0.0 (ref)	
	IYCF	66	20.9 (4.2)	0.21 (−1.13 to 1.55)	0.756	−0.65 (−1.76 to 0.46)	0.250
	WASH	83	20.0 (3.9)	−0.73 (−1.87 to 0.41)	0.209	−1.09 (−2.24 to 0.06)	0.062
	IYCF+WASH	103	22.2 (4.1)	1.48 (0.20 to 2.77)	0.024	0.65 (−0.33 to 1.63)	0.196
**MDAT social**	SOC	66	24.1 (2.1)	0.0 (ref)		0.0 (ref)	
	IYCF	66	24.7 (2.2)	0.53 (−0.00 to 1.06)	0.052	0.19 (−0.37 to 0.75)	0.510
	WASH	83	24.0 (2.4)	−0.08 (−0.70 to 0.55)	0.811	−0.23 (−0.80 to 0.34)	0.431
	IYCF+WASH	103	25.0 (2.2)	0.99 (0.49 to 1.48)	<0.001	0.61 (0.13 to 1.09)	0.013
**MacArthur Bates (CDI**)	SOC	66	56.6 (18.5)	0.0 (ref)		0.0 (ref)	
	IYCF	65	57.6 (21.3)	1.00 (−5.74 to 7.55)	0.771	−2.47 (−8.60 to 3.67)	0.431
	WASH	79	58.2 (20.1)	1.58 (−4.12 to 7.29)	0.586	−2.27 (−8.14 to 3.60)	0.448
	IYCF+WASH	99	65.1 (17.0)	8.50 (3.66 to 13.33)	0.001	6.01 (1.14 to 10.88)	0.015

	Effects comparing WASH vs non-WASH and IYCF vs non-IYCF									
	Treatment group	N	Mean (SD)	Treatment group	N	Mean (SD)	Unadjusted difference (95% CI)	P value	Adjusted* difference (95% CI)	P value
**A not B**	SOC	55	7.9 (1.3)	IYCF: no	131	7.8 (1.4)	0.0 (ref)		0.0 (ref)	
	IYCF	62	7.7 (1.3)	IYCF: yes	156	7.7 (1.3)	−0.11 (−0.42 to 0.20)	0.487	0.05 (−0.24 to 0.34)	0.745
	WASH	76	7.8 (1.5)	WASH: no	117	7.8 (1.3)	0.0 (ref)		0.0 (ref)	
	IYCF+WASH	94	7.7 (1.3)	WASH: yes	170	7.8 (1.4)	0.0 (−0.30 to 0.31)	0.994	−0.10 (−0.38 to 0.18)	0.472

Primary dichotomous outcomes	Treatment group	N	n (%)	Treatment group	N	n (%)	Unadjusted relative risk (95% CI)	P value	Adjusted relative risk (95% CI)	P value
**Self-control task (hidden**)	SOC	62	17 (27.4%)	IYCF: no	144	49 (34.0%)	1.00 (ref)		1.00 (ref)	
	IYCF	66	25 (37.9%)	IYCF: yes	168	58 (34.5%)	1.00 (0.74 to 1.34)	0.975	1.05 (0.77 to 1.45)	0.743
	WASH	82	32 (39.0%)	WASH: no	128	42 (32.8%)	1.00 (ref)		1.00 (ref)	
	IYCF+WASH	102	33 (32.4%)	WASH: yes	184	65 (35.3%)	1.08 (0.78 to 1.49)	0.631	0.97 (0.69 to 1.37)	0.878
**Self-control task (unhidden**)	SOC	61	30 (49.2%)	IYCF: no	142	79 (55.6%)	1.00 (ref)		1.00 (ref)	
	IYCF	66	35 (53.0%)	IYCF: yes	166	88 (53.0%)	0.94 (0.77 to 1.41)	0.522	0.92 (0.73 to 1.15)	0.458
	WASH	81	49 (60.5%)	WASH: no	127	65 (51.2%)	1.00 (ref)		1.00 (ref)	
	IYCF+WASH	100	53 (53.0%)	WASH: yes	181	102 (56.4%)	1.11 (0.90 to 1.36)	0.350	1.09 (0.86 to 1.38)	0.467

Secondary dichotomous language outcomes	Effects by each randomised arm compared with the SOC ARM						
	Treatment group	N	N (%)	Unadjusted relative risk (95% CI)	P	Adjusted relative risk (95% CI)	P
**Uses plurals**	SOC	66	8 (12.1%)	1.00 (ref)		1.00 (ref)	
	IYCF	66	17 (25.8%)	1.97 (0.91 to 4.27)	0.087	1.05 (0.49 to 2.22)	0.906
	WASH	83	14 (16.7%)	1.21 (0.51 to 2.88)	0.667	1.15 (0.58 to 2.26)	0.695
	IYCF+WASH	103	27 (26.2%)	2.08 (0.98 to 4.41)	0.055	1.57 (0.79 to 3.11)	0.195
**Combines two words**	SOC	66	64 (97.0%)	1.00 (ref)		1.00 (ref)	
	IYCF	66	63 (95.5%)	0.99 (0.92 to 1.06)	0.737	0.99 (0.92 to 1.05)	0.646
	WASH	83	82 (98.8%)	1.02 (0.97 to 1.07)	0.407	1.01 (0.96 to 1.07)	0.722
	IYCF+WASH	103	103 (100.0%)	1.03 (0.99 to 1.08)	0.158	1.01 (0.97 to 1.06)	0.566
**Uses imperatives**	SOC	66	49 (74.2%)	1.00 (ref)		1.00 (ref)	
	IYCF	66	46 (69.7%)	0.94 (0.78 to 1.13)	0.479	0.92 (0.75 to 1.11)	0.380
	WASH	83	57 (68.7%)	0.91 (0.81 to 1.02)	0.123	0.86 (0.71 to 1.05)	0.139
	IYCF+WASH	102	77 (75.5%)	1.00 (0.87 to 1.15)	0.973	1.01 (0.84 to 1.21)	0.940

*Scores were adjusted for the following variables: maternal baseline mid-upper arm circumference, education, employment status, CD4 count, cotrimoxazole prophylaxis and antiretroviral treatment during pregnancy, capabilities (perceived physical health and decision-making autonomy), access to improved latrine; infant low birth weight, prematurity, gender and age at assessment; season of recruitment and research nurse who carried out the assessment.

CDI, Communication Development Inventories; IYCF, infant and young child feeding; MDAT, Malawi Developmental Assessment Tool; SOC, standard-of-care; WASH, water, sanitation and hygiene.

### ​MacArthur Bates CDI vocabulary and grammar checklists

Compared with SOC, children in the IYCF +WASH arm had higher MacArthur Bates CDI vocabulary scores (+8.5 words; 95% CI 3.7 to 13.3), corresponding to an increase of 0.44 SD. Twice as many children in the IYCF +WASH arm reportedly used plurals (RR 2.08; 95% CI 0.98 to 4.41), although this difference was no longer significant in the adjusted analysis. There was no evidence of an effect of IYCF +WASH on the proportion of children combining two words or using the progressive tense. There was no evidence that either the IYCF or WASH interventions, when implemented alone, impacted any component of the MacArthur Bates CDI score (table 3).

### ​Object permanence and self-control

There was no interaction between interventions for either test, so the IYCF arms were compared with non-IYCF arms, and the WASH arms were compared with non-WASH arms. There was no evidence of differences between intervention groups in object permanence or in the proportion of children with self-control (table 3). Inferences were similar in adjusted analyses.

### ​Sensitivity and subgroup analyses

When effects of the interventions were restricted to children confirmed as HEU (ie, removing children who were HIV-positive and HIV-unknown at 18 months of age), overall findings were similar (table 4). There was a significant interaction between child gender and randomised interventions for the total MDAT score. Results stratified by gender showed that among girls, those in the combined IYCF+WASH arm had significantly higher motor, language and social scores than those in the SOC arm. Among boys, those in the combined IYCF+WASH arm had significantly higher language and social scores than those in the SOC arm, but there was no evidence of an intervention effect on motor scores (online supplementary table 2).

10.1136/bmjgh-2019-001718.supp3 Supplementary data

**Table 4:** Effect of WASH and IYCF inventories on early child development at 24 months among HIV-exposed uninfected children

Primary continuous outcomes	Effects by each randomised arm compared with the SOC arm				
	Treatment group	N	Mean (SD)	Unadjusted difference between means (95% CI)	P value
**MDAT total score**	SOC	63	90.7 (8.1)	0.0 (ref)	
	IYCF	63	91.8 (8.6)	1.26 (–1.40 to 3.91)	0.353
	WASH	79	89.6 (9.2)	−0.81 (–3.30 to 1.68)	0.525
	IYCF+WASH	95	95.5 (9.0)	5.70 (3.00 to 8.39)	<0.001
**MDAT gross motor**	SOC	63	23.0 (2.6)	0.0 (ref)	
	IYCF	63	23.5 (2.8)	0.51 (–0.40 to 1.41)	0.271
	WASH	79	22.7 (3.3)	−0.27 (–1.06 to 0.52)	0.500
	IYCF+WASH	95	24.2 (3.3)	1.21 (0.37 to 2.06)	0.005
**MDAT fine motor**	SOC	63	22.9 (2.4)	0.0 (ref)	
	IYCF	63	22.7 (3.5)	−0.24 (–1.33 to 0.85)	0.662
	WASH	79	22.9 (2.7)	−0.03 (–0.80 to 0.75)	0.946
	IYCF+WASH	95	23.8 (2.6)	0.88 (0.11 to 1.65)	0.024
**MDAT language**	SOC	63	20.7 (3.8)	0.0 (ref)	
	IYCF	63	21.0 (4.1)	0.29 (–1.03 to 1.61)	0.669
	WASH	79	20.1 (3.9)	−0.64 (–1.80 to 0.53)	0.284
	IYCF+WASH	95	22.4 (4.0)	1.78 (0.45 to 3.11)	0.009
**MDAT social**	SOC	63	24.1 (2.1)	0.0 (ref)	
	IYCF	63	24.7 (2.2)	0.55 (0.00 to 1.09)	0.050
	WASH	79	24.0 (2.4)	−0.04 (–0.65 to 0.57)	0.896
	IYCF+WASH	95	25.1 (2.2)	1.08 (0.56 to 1.60)	<0.001
**MacArthur Bates (CDI**)	SOC	63	56.9 (18.3)	0.0 (ref)	
	IYCF	62	58.4 (20.7)	1.70 (–4.84 to 8.24)	0.610
	WASH	75	58.4 (18.9)	1.65 (–3.57 to 6.87)	0.535
	IYCF+WASH	91	66.0 (15.9)	9.36 (4.62 to 14.10)	<0.001
	**Effects comparing WASH versus non-WASH and IYCF versus non-IYCF**				

	Effects comparing WASH versus non-WASH and IYCF versus non-IYCF							
	Treatment group	N	Mean (SD)	Treatment group	N	Mean (SD)	Unadjusted difference between means (95% CI)	P value
**A not B**	SOC	52	7.8 (1.3)	IYCF: no	124	7.8 (1.4)	0.0 (ref)	
	IYCF	59	7.6 (1.3)	IYCF: yes	145	7.7 (1.3)	−0.11 (–0.43 to 0.22)	0.529
	WASH	72	7.8 (1.5)	WASH: no	111	7.7 (1.3)	0.0 (ref)	
	IYCF+WASH	86	7.7 (1.3)	WASH: yes	158	7.8 (1.4)	0.03 (–0.30 to 0.35)	0.878

Primary dichotomous outcomes	Treatment group	N	n (%)	Treatment group	N	n (%)	Unadjusted relative risk (95% CI)	P value
**Self-control task (hidden**)	SOC	59	16 (27.1%)	IYCF: no	137	47 (34.3%)	1.00 (ref)	
	IYCF	63	24 (38.1%)	IYCF: yes	157	53 (33.8%)	1.07 (0.77 to 1.49)	0.698
	WASH	78	31 (39.7%)	WASH: no	122	40 (32.8%)	1.00 (ref)	
	IYCF+WASH	94	29 (30.9%)	WASH: yes	172	60 (34.9%)	0.97 (0.71 to 1.32)	0.839
**Self-control task (unhidden**)	SOC	58	27 (46.6%)	IYCF: no	135	74 (54.8%)	1.00 (ref)	
	IYCF	63	34 (54.0%)	IYCF: yes	155	83 (53.5%)	0.94 (0.77 to 1.15)	0.563
	WASH	77	47 (61.0%)	WASH: no	121	61 (50.4%)	1.00 (ref)	
	IYCF+WASH	92	49 (53.3%)	WASH: yes	169	96 (56.8%)	1.14 (0.92 to 1.41)	0.219

Secondary dichotomous language outcomes	Effects by each randomised arm compared with the SOC ARM			
	Treatment group	N	Unadjusted Relative Risk (95% CI)	P value
**Uses plurals**	SOC	63	1.00 (ref)	
	IYCF	63	1.87 (0.85 to 4.12)	0.118
	WASH	79	1.14 (0.48 to 2.68)	0.764
	IYCF+WASH	95	2.18 (1.03 to 4.60)	0.041
**Combines two words**	SOC	63	1.00 (ref)	
	IYCF	63	1.00 (0.94 to 1.06)	0.990
	WASH	79	1.02 (0.97 to 1.07)	0.446
	IYCF+WASH	95	*	–
**Uses imperatives**	SOC	63	1.00 (ref)	
	IYCF	63	0.96 (0.80 to 1.15)	0.645
	WASH	79	0.93 (0.80 to 1.09)	0.387
	IYCF+WASH	95	1.01 (0.87 to 1.17)	0.869

*All the children in the IYCF+WASH arm combined two words.

CDI, Communicative Development Inventories; IYCF, infant and young child feeding; MDAT, Malawi Developmental Assessment Tool;SOC, standard-of-care; WASH, water, sanitation and hygiene.

## Discussion

In this analysis of children born to HIV-positive mothers in rural Zimbabwe, a package combining IYCF and WASH interventions significantly improved motor, language and cognitive development at 2 years of age compared with those receiving enhanced SOC. The same IYCF and WASH interventions when delivered individually (rather than as a combined package) had no impact on ECD at 2 years of age. These findings are contrary to previously reported findings from the SHINE trial among a larger group of 3686 HIV-unexposed children, in whom the IYCF and WASH interventions, delivered either alone or together, had no meaningful impact on ECD. We suggest from these collective findings that HIV exposure in early life is a distinct challenge and that HEU children may be particularly responsive to a package of interventions to improve neurocognitive development.

The SHINE trial was designed to evaluate the effects of IYCF and WASH on linear growth and haemoglobin, which are both associated with child development. In the SHINE trial, among children born to HIV-negative mothers, the IYCF intervention increased LAZ by 0.16 (95% CI 0.08 to 0.23) and haemoglobin by 2.03 (95% CI 1.28 to 2.79) g/L at 18 months of age, while the WASH intervention had no effect on these outcomes.^8^ Among 668 children born to HIV-positive mothers, the effects of the IYCF intervention exceeded those seen in the HIV-unexposed group, increasing mean Length for Age Z score (LAZ) by 0.26 (95% CI 0.09 to 0.43) and haemoglobin by 2.9 (95% CI 0.9 to 4.9) g/L, while the WASH intervention had no effect on these outcomes.^9^ In both analyses, implementing WASH together with IYCF had no additional impact on stunting or anaemia compared with delivering IYCF alone. In the current analysis, HIV-exposed children randomised to a package of combined IYCF +WASH interventions had significantly improved motor, social and language development compared with HIV-exposed children receiving SOC interventions; by contrast, those randomised to receive either the IYCF or WASH intervention alone had similar ECD scores to control children. It is difficult to explain why combining IYCF and WASH improved neurodevelopment in HIV-exposed children when we found little evidence of benefit from either intervention delivered alone. We did not see this level of synergy between the interventions in HIV-unexposed children, although the study was not powered to distinguish between interactions in these two subgroups. However, among the HIV-exposed children, we found consistent, statistically significant improvements across multiple tests of global child development. These included specific motor, social and language scores, which exhibited substantial magnitudes of effect between IYCF +WASH and SOC of 0.4–0.5 SD.^18 19^ These effect sizes effects are considerable for child development at this age.

We have previously reported that HEU children had evidence of modestly reduced developmental scores compared with HIV-unexposed children in SHINE,^20^ but we suggest from the results of this current study that they may also be more responsive to interventions (online supplementary table 3). The IYCF intervention was designed to improve nutrient intake during a critical period of growth and brain development, while the WASH intervention was designed to reduce exposure to pathogens and to prevent a subclinical inflammatory disorder of the gut termed EED. Perhaps ECD improvements in these vulnerable children are only realised when gut health and nutrient requirements are both addressed simultaneously. A healthier intestinal milieu may facilitate absorption and prevent wastage of nutrients from the IYCF intervention, or modulate the microbiota–gut–brain axis to improve neurodevelopment. Further planned laboratory studies of EED biomarkers, pathogen carriage and growth hormone activity will help to address the underlying pathways. We anticipated that the infant feeding intervention alone would lead to improvements in ECD, since IYCF reduced stunting at 18 months of age, but IYCF alone had no evidence of benefit for neurodevelopment. There was no evidence for a synergistic effect of IYCF and WASH on linear growth at 18 months of age, although children randomised to this group did have a larger head circumference (mean Z-score −0.38, compared with SOC −0.55, IYCF −0.51 and WASH −0.53). It is, therefore, plausible that HIV-exposed children prioritise head growth over linear growth; however, this difference was already apparent from as early as 3 months of age. We are, therefore, uncertain whether our findings reflect a true effect of the combined intervention on ECD, and we also need to consider alternative explanations for these results.

10.1136/bmjgh-2019-001718.supp4 Supplementary data

First, it is possible that there was residual confounding in this substudy. Although our findings remained consistent after adjusting for known confounders, there may have been imbalances in unmeasured factors at baseline. This study was nested within a larger randomised controlled trial, where children were identified and offered enrolment during the period of this substudy if they met strict age criteria. There were some differences in rates of enrolment into the ECD study across the treatment arms which may have created bias. There was balance on baseline demographics across trial arms for those who were enrolled in the substudy, which increases our confidence in the internal validity of our findings. Overall, there was similarity between those who were enrolled and not enrolled from the main SHINE trial; however, there were some differences that may influence external validity, such as a higher rate of institutional delivery and a shorter walk-time to water in the IYCF+WASH arm among those assessed for ECD compared with those not assessed.

Given our incomplete understanding of the interlinking pathways between HIV exposure and neurodevelopment, it is unlikely that all factors were accounted for in our analyses. For example, it is possible that children in the IYCF-only group had more risk factors (constraints at the maternal, child and household level) for poor ECD which we have not been able to evaluate compared with other groups so that the benefits of the IYCF intervention were only observed in the combined IYCF +WASH group. These risk factors may be related to the caregiver’s capacity to provide responsive caregiving, in turn, affecting the child’s development. Second, the households in the combined IYCF +WASH group received more trial inputs than the IYCF-only or WASH-only groups. In designing SHINE, we were careful to ensure that all families received the same number of VHW contacts; however, visits in the IYCF +WASH arm were longer and households in the combined IYCF +WASH group received more hardware (latrine and tippy taps) and commodities (chlorine, soap and SQ-LNS) than the IYCF-only or WASH-only groups. Both the IYCF and WASH modules may have increased the interactions between mother and child (eg, washing child’s hands and providing responsive feeding) and given the strong evidence linking positive maternal–child interaction and ECD outcomes, it is possible that these combined interventions in some way enabled an increase in interaction time to impact ECD scores. In addition, the positive impact of receiving inputs which included longer visits as well as the material goods (a latrine, tippy tap and nutritional supplements) may have positively impacted maternal mental health and well-being which may have influenced ECD outcomes.^21 22^ Quality of caregiving and responsivity plays a critical role within ECD intervention studies and it may be that the additional inputs and VHW time that were provided in the combined group with both curricula improved maternal well-being and enabled increased responsivity more in the vulnerable HIV-positive women than in HIV-negative women, and this, in turn, improved ECD in their children. In other words, the ‘double’ training pack received from VHWs along with the additional inputs may have enabled mothers to be more responsive to the health and developmental needs of their HEU children. This may also reflect the fact that we saw a specific neurodevelopmental profile change in the areas of language and fine motor development (both closely linked to responsive caregiving) than in the gross motor and social components of development. With this in mind, a package of interventions which enhances quality caregiving and responsivity as much as possible may be what is needed to meaningfully impact ECD.

We found a significant interaction between child gender and trial intervention. Among girls, compared with the SOC arm, those in the combined IYCF+WASH arm had significantly higher motor, language and social scores. Among boys, compared with the SOC arm, those in the combined IYCF+WASH arm had significantly higher language and social scores, but there was no evidence of an effect on motor scores. Among children in the SOC arm, boys generally had poorer language and social scores than girls, whereas motor scores were similar; it is possible that whereas girls generally responded to IYCF+WASH interventions in all domains, boys only responded when developmental delays were more pronounced.

This analysis has several strengths. To our knowledge, this is the first intervention trial evaluating ECD outcomes in HIV-exposed children, who are an expanding population in regions with high ongoing antenatal HIV prevalence. By using several different developmental assessment tools, we were able to thoroughly assess ECD across a broad range of domains. We adapted our assessment tools for use in rural Zimbabwe, and undertook extensive piloting, quality control and standardisation. We were able to report the findings specifically for HEU children, who now comprise the vast majority of children born to HIV-infected mothers. However, an important limitation of this study is that SHINE was originally designed to assess the effects of IYCF and WASH interventions on stunting and anaemia, and was powered to evaluate these outcomes in HIV-unexposed children; the ECD component was a substudy of the trial, and the current findings focus only on HIV-exposed children. Further studies are needed to confirm our findings in other populations.

In summary, HIV-exposed children randomised to a combined intervention of improved ICYF and improved WASH had significant improvements in ECD at 2 years of age, while those receiving either the IYCF or WASH intervention alone had no evidence of ECD benefit. Importantly, combining the IYCF and WASH interventions closed the developmental gap between HEU and HIV-unexposed children. Although the mechanisms that underlie the synergistic effects of IYCF and WASH on ECD in HIV-exposed children remain unclear, our study implicates nutrition and WASH as contributing factors in the neurocognitive development of children exposed to HIV in early life that warrant further study. Thus, despite having poorer health outcomes than HIV-unexposed children in the absence of any interventions, we suggest from the results of this study, that HEU children may be more responsive to public health interventions. The interventions we provided combined WASH improvements with nutritional support, which goes beyond what current PMTCT programmes provide. Combining these approaches with specific nurturing care and early education interventions, in line with the Nurturing Care Framework strategy, may bring additional benefits to improve human capital in this expanding global population.
