# Supplementary material for: Effects of improved complementary feeding and improved water, sanitation and hygiene on early child development among HIV-exposed children: substudy of a cluster randomised trial in rural Zimbabwe
Source: BMJ Glob Health. 2020 Jan 13;5(1):e001718. doi: 10.1136/bmjgh-2019-001718 (PMC7042608; doi:10.1136/bmjgh-2019-001718)
Supplement: Supplementary data [file bmjgh-2019-001718supp001.pdf]

SUPPLEMENTARY METHODS

Further details of interventions

Standard of Care (SOC) Intervention: Village Health Workers were trained through the Ministry of Health and Child Care curriculum, which instructs VHWs to visit pregnant women and infants frequently, although the precise content or purpose of each visit is not specified. Consequently the SHINE SOC intervention was designed to standardize the number of visits (3 antenatal and 12 postnatal visits) and the content of primary health care messages across treatment arms. Four of these visits promoted exclusive breastfeeding (EBF) from birth to 6 months using modules designed to overcome contextual barriers identified in formative work. Other SOC modules include prevention of mother-to-child HIV transmission (PMTCT), antenatal care, hospital-based delivery, family planning and immunizations.

WASH Intervention: Within 6 weeks of enrolment (~20 weeks gestation) into the WASH and WASH+IYCF arms of the trial, a Blair Ventilated Improved Pit (VIP) Latrine was constructed at the participant's household and two 'Tippy Tap' hand-washing stations (locally manufactured, and adapting the model piloted by the Kenya WASH Benefits trial) were installed near the latrine and kitchen; Figure S1.

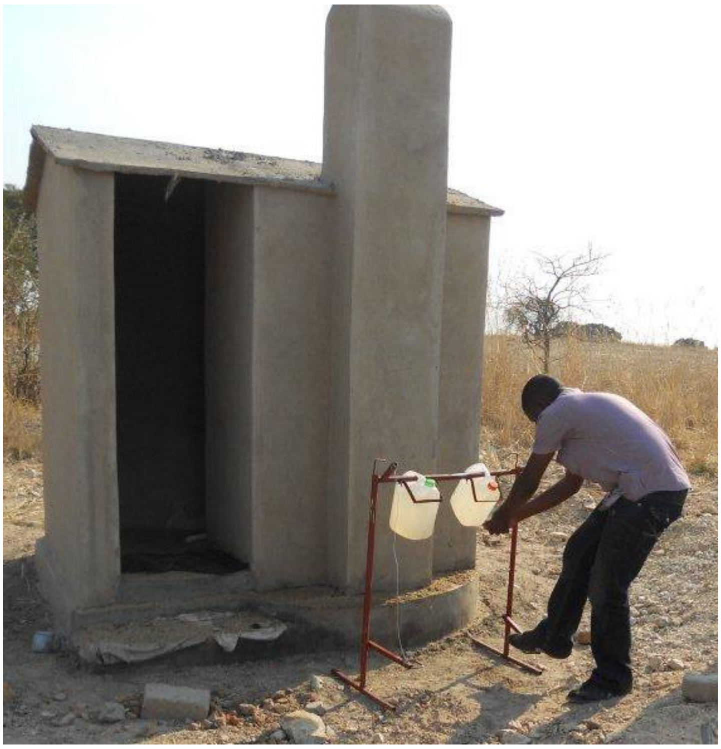

Figure S1: Blair VIP latrine and Tippy Tap

WASH Modules 1 (delivered at 24 gestational weeks) and 2 (32 gestational weeks) promoted safe disposal of faeces, and hand-washing with soap after faecal contact and before food preparation and eating, respectively. Our intention was for the baby to be born into a household in which latrine use and household hand-washing behaviours were normalised and habitual. WASH Module 3 (protecting babies from faecal ingestion during exploratory play) was delivered when the baby was 2 months old; a washable 2.8m x 3.0m locally manufactured mat and plastic play yard (North States, Minneapolis MN) were provided at 2 months and 6 months, respectively; Figure S2.

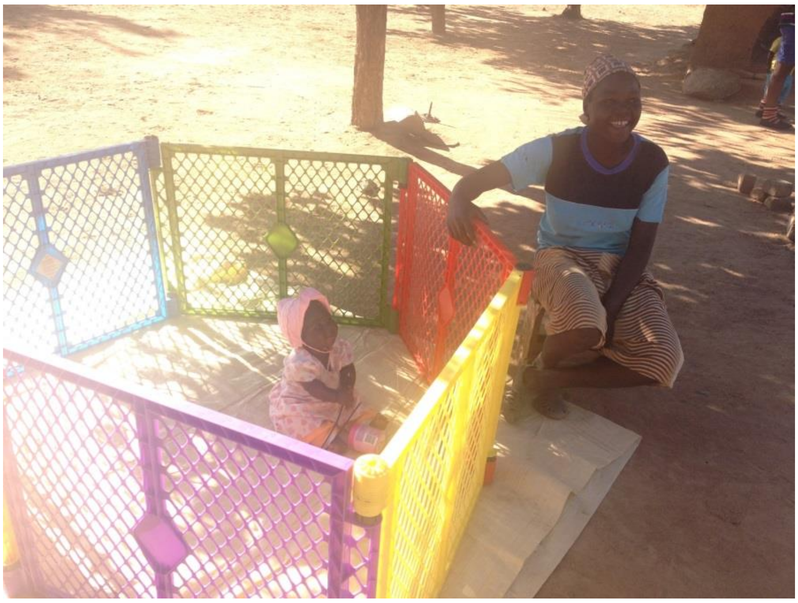

Figure S2: SHINE mat and play yard

WASH Module 4 (treat all drinking water given to babies after 6 months of EBF) was delivered at 4 months of age, along with point-of-use chlorination (WaterGuard: a dilute sodium hypochlorite solution, manufactured locally by Nelspot). Liquid soap and Water Guard were regularly replenished from time of introduction (Module 2 and 5, respectively) until the infant was 18 months old. WASH Module 5, delivered at 5 months of age, stressed the importance of freshly preparing or fully reheating all foods fed to infants. A review module was delivered at 12 months.

IYCF Intervention: IYCF Module 1 (delivered at 5 months) linked good infant feeding to child growth, health, and intelligence. IYCF Module 2 (6 months) promoted feeding nutrient-dense food, including 20 g per day of the lipid-based nutrient supplement (LiNS) developed by the International Lipid-Based Nutrients Supplements Project, provided monthly when the baby was 6 to 18 months of age. Module 3 (7 months) was a participatory cooking

demonstration in which any available household food was prepared and fed to the baby, stressing three messages from formative research: 1) an infant can eat any food that an adult eats; 2) food should be ground so that the infant can swallow and digest it; 3) food that is locally available is important for the infant. Module 4 (8 months) promoted responsive feeding during illness, Module 5 (9 months) promoted diet diversity, and a review module was delivered at 12 months.
