# Supplementary material for: Effects of improved complementary feeding and improved water, sanitation and hygiene on early child development among HIV-exposed children: substudy of a cluster randomised trial in rural Zimbabwe
Source: BMJ Glob Health. 2020 Jan 13;5(1):e001718. doi: 10.1136/bmjgh-2019-001718 (PMC7042608; doi:10.1136/bmjgh-2019-001718)
Supplement: Supplementary data [file bmjgh-2019-001718supp002.pdf]

Supplementary Table 1: Baseline characteristics of mothers and infants who were enrolled and not enrolledinto the early child development sub-study

	SOC			IYCF			WASH			WASH plus IYCF		
**Baseline characteristics**	**Enrolled**	**Not Enrolled**	***P Value***	**Enrolled**	**Not Enrolled**	***P Value***	**Enrolled**	**Not Enrolled**	***P Value***	**Enrolled**	**Not Enrolled**	***P Value***
Mothers	68	78		67	77		81	99		102	84	
Infants	68	78		68	79		83	101		104	86	
Mothers completing baseline visit[1]	67	77		65	76		80	99		101	83	
**Household characteristics**												
Median number of occupants (IQR)	4.5 (3,6)	4 (3,5)	0.111	4 (3,6)	4 (3,6)	0.93	5 (3,6)	4 (3,6)	0.60	4 (3,6)	4 (3,6)	0.62
Wealth Quintile[2]												
1 (Lowest)	16/66 (24.2%)	22/77 (28.5%)		14/65 (21.5%)	22/76 (29.0%)		20/78 (25.6%)	25/99 (25.3%)		28/100 (28.0%)	20/82 (24.4%)	
2	15/66 (22.7%)	27/77 35.1(%)		13/65 (20.0%)	12/76 (15.8%)		15/78 (19.3%)	24/99 (24.2%)		18/100 (18.0%)	26/82 (31.7%)	
3	13/66 (19.7%)	18/77 (23.4%)	0.0768	13/65 (20.0%)	18/76 (23.7%)	0.564	19/78 (24.4%)	16/99 (16.2%)	0.49	18/100 (18.0%)	14/82 (17.1%)	0.28
4	8/66 (12.1%)	6/77 (7.8%)		17/65 (26.2%)	15/76 (19.7%)		11/78 (14.1%)	13/99 (13.1%)		17/100 (17.0%)	7/82 (8.5%)	
5 (Highest)	14/66 (21.2%)	4/77 (5.2%)		8/65 (12.3%)	9/76 (11.8%)		13/78 (16.7%)	21/99 (21.2%)		19/100 (19.0%)	15/82 (18.3%)	
Electricity												
Connected to power grid	3/66 (3.5%)	2/77 (2.6%)	0.524	3/65 (4.6%)	0/76 (0.0%)	0.0000	0/78 (0.0%)	3/99 (3.0%)	0.0000	4/100 (4.0%)	3/83 (3.6%)	0.891
Other power:												
Uses a generator	2/66 (3.0%)	1/77 (1.3%)		3/65 (4.6%)	3/76 (4.0%)		0/78 (0.0%)	2/99 (2.0%)		3/100 (3.0%)	2/83 (2.4%)	
Uses solar power	45/66 (68.2%)	42/77 (54.6%)	0.074	44/65 (67.7%)	52/76 (68.4%)	0.98	56/78 (71.8%)	62/99 (62.6%)	0.0000	64/100 (60.0%)	53/83 (63.9%)	0.97
No electricity	19/66 (28.8%)	34/77 (44.2%)		18/65 (27.7%)	21/76 (27.6%)		22/78 (28.2%)	35/99 (35.4%)		33/100 (33.0%)	28/83 (33.7%)	
Sanitation												
Household members defecate in the open												
Any latrine at household	18/66 (27.3%)	24/77 (31.2%)	0.621	28/62 (45.2%)	25/75 (33.3%)	0.176	29/77 (37.7%)	45/99 (45.5%)	0.319	37/96 (38.5%)	31/82 (37.8%)	0.926
Improved latrine at household	17/66 (25.8%)	18/77 (23.4%)	0.739	24/62 (38.7%)	23/75 (30.7%)	0.391	25/77 (32.5%)	41/99 (41.4%)	0.211	36/96 (37.5%)	24/81 (29.6%)	0.254

Improved latrine with well-trodden path	14/66 (21.2%)	11/76 (14.5%)	0.327	21/62 (33.9%)	18/75 (24.0%)	0.214	23/77 (29.9%)	29/99 (30.0%)	0.921	25/96 (26.0%)	17/81 (21.0%)	0.449
Improved latrine with well-trodden path and not shared	12/64 (18.8%)	7/73 (9.6%)	0.098	19/60 (31.7%)	17/100 (17.0%)	0.289	21/76 (27.6%)	26/97 (26.8%)	0.890	23/94 (24.5%)	12/76 (15.8%)	0.157
Water												
Main source of household drinking water is improved	41/66 (62.1%)	46/77 (59.7%)	0.766	36/62 (58.1%)	46/75 (61.3%)	0.710	44/77 (57.1%)	56/97 (57.7%)	0.928	59/96 (61.5%)	52/82 (63.4%)	0.782
Treat drinking water to make it safer	13/64 (20.3%)	7/76 (9.2%)	0.107	11/62 (17.7%)	8/72 (11.1%)	0.324	10/77 (13.0%)	6/96 (6.3%)	0.173	13/96 (13.5%)	9/82 (11.0%)	0.601
Median one-way walk time to fetch water (IQR), min	10 (5,20)	15 (5,30)	0.501	9 (3,15)	10 (5,15)	0.809	10 (5,15)	10 (5,30)	0.226	8.5 (5,20)	15 (5,20)	0.015
Mean water volume collected per person in past 24 h (SD), L	8.7 (5.0)	8.6 (4.8)	0.922	9.4 (6.7)	10.4 (6.9)	0.392	9.2 (6.6)	9.7 (7.7)	0.777	10.4 (7.5)	8.7 (5.6)	0.157
Hygiene												
Handwashing station at household	3/54 (5.6%)	1/69 (1.5%)	0.238	4/58 (6.5%)	4/71 (5.6%)	0.858	11/74 (14.9%)	19/95 (20.0%)	0.426	15/91 (16.5%)	7/78 (9.0%)	0.148
Handwashing station with water	1/54 (1.9%)	1/69 (1.5%)	0.865	0/60 (0.0%)	1/69 (1.5%)	0.000	4/74 (5.4%)	4/94 (4.3%)	0.748	2/91 (2.2%)	2/78 (2.6%)	0.878
Handwashing station with water and rubbing agent	0/54 (0.0%)	0/69 (0.0%)	-	0/60	0/69 (0.0%)	-	0/74 (0.0%)	0/94 (0.0%)	-	1/90 (1.1%)	0/78 (0.0%)	-
Improved floor[3]	28/68 (41.2%)	24/78 (30.8%)	0.183	32/67 (47.8%)	33/77 (42.9%)	0.564	32/81 (39.5%)	48/99 (48.5%)	0.220	42/%)	38/84 (45.2%)	0.565
Median number of chickens (IQR)	4 (1, 8)	4 (0, 10)	0.986	5.5 (2, 10.5)	4 (0, 8)	.045	5 (3, 8)	4.5 (2, 10)	0.775	4 (1, 8)	5 (0, 8)	0.711
Livestock observed inside home	28/65 (43.1%)	27/77 (35.1%)	0.332	19/65 (29.2%)	23/75 (30.7%)	0.858	34/78 (43.6%)	33/97 (34.0%)	0.165	33/99 (33.3%)	25/81 (30.9%)	0.713
Faeces observed in yard	25/68 (36.8%)	28/78 (35.9%)	0.919	17/67 (25.4%)	22/77 (28.6%)	0.679	26/81 (32.1%)	26/99 (26.35)	0.401	17/102 (16.7%)	23/84 (27.4%)	0.072
Diet quality and food security												
Household meets minimum Dietary Diversity [4]	21/55 (38.2%)	25/66 (37.9%)	0.967	22/57 (38.6%)	27/65 (41.5%)	0.772	24/73 (32.9%)	31/84 (36.9%)	0.610	37/83 (44.6%)	28/70 (40.0%)	0.533
Median Coping Strategies Index score[5] (IQR)	4 (0, 9.5)	3 (0, 9)	0.654	2 (0, 15)	2 (0, 14)	0.746	3 (0, 11)	0 (0, 9)	0.216	1 (0, 9)	3 (0, 10.5)	0.289
**Maternal characteristics**												
Mean age (SD), years	30.8 (6.1)	29.3 (6.4)	0.18	30.9 (6.4)	28.3 (5.8)	0.0169	30.4 (6.5)	27.3 (5.6)	0.0007	30.9 (5.8)	28.4 (6.0)	0.0017
Mean height (SD), cm	161.2 (7.2)	161.2 (5.6)	0.90	160.2 (6.2)	160.1 (5.8)	0.89	160.5 (6.3)	160.4 (6.5)	0.94	159.0 (6.2)	159.8 (5.3)	0.33

Mean mid-upper arm circumference (SD), cm	26.8 (3.5)	26.1 (3.0)	0.125	26.3 (3.3)	26.1 (2.4)	0.669	26.4 (2.9)	26.4 (2.6)	0.99#	26.4 (2.9)	25.9 (2.6)	0.097
Mean years of completed schooling (SD), years	9.5 (1.9)	9.4 (2.1)	0.852	9.0 (2.1)	9.0 (2.3)	0.946	8.7 (2.3)	9.1 (2.2)	0.137	9.3 (1.7)	9.2 (2.0)	0.748
Positive microscopy for *Schistosoma haematobium*	6/67 (9.0%)	6/68 (8.8%)	0.98	4/64 (6.3%)	5/72 (6.9%)	0.858	12/78 (15.4%)	8/90 (8.9%)	0.237	9/101 (8.9%)	10/74 (13.5%)	0.409
Median parity (IQR)	3 (1, 3)	2 (1, 3)	0.041	2 (1, 3)	2 (1, 3)	0.605	3 (2, 4)	2 (1, 3)	0.002	2 (1, 3)	2 (1, 3)	0.839
Married	61/64 (95.3%)	67/72 (93.1%)	0.587	59/63 (93.7%)	71/75 (94.7%)	0.835	71/73 (97.3%)	90/96 (93.8%)	0.365	85/92 (92.0%)	75/80 (93.8%)	0.774
Employed	5/65 (7.7%)	9/76 (11.8%)	0.393	5/65 (7.7%)	8/76 (10.5%)	0.563	8/78 (10.3%)	12/99 (12.1%)	0.690	8/100 (8.0%)	3/83 (3.6%)	0.266
Religion:												
Apostolic	34/68 (50.0%)	31/78 (39.7%)		32/67 (47.8%)	29/77 (37.7%)		34/81 (42.0%)	47/99 (47.5%)		46/102 (45.1%)	40/84 (47.6%)	
Other Christian	25/68 (36.8%)	34/78 (43.6%)	0.37	25/67 (37.3%)	38/77 (49.4%)	0.27	30/81 (37.0%)	38/99 (38.4%)	0.50	41/102 (40.2%)	35/84 (41.7%)	0.72
Other	9/68 (13.2%)	13/78 (16.7%)		10/67 (14.9%)	10/77 (13.0%)		17/81 (21.0%)	14/99 (14.1%)		15/102 (14.7%)	9/84 (10.7%)	
**Infant characteristics**												
Female	33/68 (48.5%)	37/78 (47.4%)	0.90	32/68 (47.1%)	39/79 (49.4%)	0.77	40/83 (48.2%)	51/101 (50.1%)	0.77	55/104 (52.9%)	49/86 (57.0%)	0.66
Mean birth weight (SD), kg	3.03 (0.48)	3.04 (0.50)	0.819	2.93 (0.49)	3.03 (0.45)	0.243	3.04 (0.51)	2.99 (0.47)	0.532	3.00 (0.50)	2.94 (0.45)	0.237
Birth weight <2500 g	8/67 (11.9%)	8/69 (11.6%)	0.953	10/66 (15.2%)	7/72 (9.7%)	0.423	6/78 (7.7%)	13/86 (15.1%)	0.180	11/101 (10.9%)	9/71 (12.7%)	0.666
Institutional delivery	59/64 (92.2%)	53/70 (75.7%)	0.0078	55/67 (82.1%)	61/73 (83.6%)	0.81	69/81 (85.2%)	78/90 (86.7%)	0.757	87/94 (92.6%)	57/77 (74.0%)	0.0009
Vaginal delivery	62/66 (93.9%)	66/70 (94.3%)	0.931	58/66 (87.9%)	70/72 (97.2%)	0.0619	78/82 (95.1%)	82/92 (89.1%)	0.1130	92/100 (92.0%)	68/77 (88.3%)	0.394

[1] Baseline variables presented for mothers who had live births; maternal and household data were collected about 2 weeks after consent (~14 weeks gestation); this gap created opportunity for loss to follow-up between consent and baseline, thus the number of mothers completing baseline visit is smaller than the number of mothers with live births. Baseline for infants was at birth. Values are %, unless noted. For variables where [n] is not stated, <3% of data are missing based on number of baseline visits completed.

[2] Chasekwa B, Maluccio JA, Ntozini R, Moulton LH, Wu F, Smith LE, et al. Measuring wealth in rural communities: Lessons from the Sanitation, Hygiene, Infant Nutrition Efficacy (SHINE) trial. PLoS ONE. 2018; 13(6): e0199393.

[3] Improved floor defined as concrete, brick, cement, or tile. Unimproved floor defined as mud, earth, sand, or dung.

[4] FAO, FHI 360. Minimum Dietary Diversity for Women: A Guide for Measurement. Rome: FAO. 2016.

[5] Maxwell D, Watkins B, Wheeler R, Collins G. The Coping Strategy Index: A tool for rapid measurement of household food security and the impact of food aid programs in humanitarian emergencies. CARE and WFP, Nairobi. 2003.

SD: standard deviation; IQR: interquartile range; MUAC: Mid-upper arm circumference
