# Supplementary material for: Effects of improved complementary feeding and improved water, sanitation and hygiene on early child development among HIV-exposed children: substudy of a cluster randomised trial in rural Zimbabwe
Source: BMJ Glob Health. 2020 Jan 13;5(1):e001718. doi: 10.1136/bmjgh-2019-001718 (PMC7042608; doi:10.1136/bmjgh-2019-001718)
Supplement: Supplementary data [file bmjgh-2019-001718supp003.pdf]

Supplementary Table 2: Effect of WASH and IYCF interventions on Early Child Development outcomes at 24 months in female and male children

CONTINUOUS OUTCOMES	Treatment group	Female children		Male children		Difference between means (95% CI)	*P*
		N	Mean score (SD)	N	Mean score (SD)		
Malawi Developmental Assessment (MDAT) Tool	SOC	32	90.2 (8.7)	34	91.7 (7.7)	1.46 (-2.68, 5.59)	0.490
	IYCF	30	91.6 (9.7)	36	91.8 (7.7)	0.26 (-4.18, 4.70)	0.909
	WASH	40	89.4 (9.3)	43	89.9 (9.1)	0.23 (-2.61, 3.07)	0.872
	IYCF+WASH	55	93.3 (7.5)	48	97.1 (9.9)	3.92 (-0.35, 8.18)	0.072
MacArthur Bates CDI (Vocabulary)	SOC	32	61.7 (16.0)	34	51.7 (19.6)	8.88 (0.64, 17.1)	0.035
	IYCF	30	60.2 (22.5)	35	55.3 (20.3)	4.75 (-3.09, 12.6)	0.235
	WASH	39	56.8 (20.5)	40	59.4 (20.0)	-2.56 (-10.5, 5.35)	0.526
	IYCF+WASH	52	67.8 (15.5)	47	62.0 (18.2)	5.85 (-2.10, 13.8)	0.149
A-not-B Test (object permanence)	SOC	28	7.9 (1.2)	27	7.9 (1.5)	-0.03 (-0.66, 0.59)	0.920
	IYCF	30	7.7 (1.3)	32	7.6 (1.4)	-0.01 (-0.63, 0.66)	0.972
	WASH	37	7.7 (1.8)	39	7.9 (1.2)	-0.14 (-0.78, 0.49)	0.656
	IYCF+WASH	52	7.8 (1.3)	42	7.7 (1.3)	0.17 (-0.39, 0.74)	0.546
**DICHOTOMOUS OUTCOMES**	**Treatment group**	**N**	**n (%)**	**N**	**n (%)**	**Unadjusted Relative Risk (95% CI)**	***P***
Self-control Task (Hidden)	SOC	30	25 (55.6%)	32	20 (44.4%)	1.33 (0.99, 1.79)	0.058
	IYCF	30	17 (41.5%)	36	24 (58.5%)	0.85 (0.59, 1.22)	0.378
	WASH	39	23 (46.0%)	43	27 (54.0%)	0.94 (0.70, 1.26)	0.672
	IYCF+WASH	54	37 (53.6%)	48	32 (46.4%)	1.03 (0.73, 1.44)	0.874
Self-control Task (Unhidden)	SOC	30	21 (67.7%)	31	10 (32.3%)	2.23 (1.32, 3.78)	0.003
	IYCF	30	12 (38.7%)	36	19 (61.3%)	0.76 (0.46, 1.25)	0.280
	WASH	38	17 (53.1%)	43	15 (46.9%)	1.26 (0.75, 2.09)	0.380
	IYCF+WASH	52	26 (55.3%)	48	21 (44.7%)	1.11 (0.76, 1.62)	0.593
