# Supplementary material for: Effects of improved complementary feeding and improved water, sanitation and hygiene on early child development among HIV-exposed children: substudy of a cluster randomised trial in rural Zimbabwe
Source: BMJ Glob Health. 2020 Jan 13;5(1):e001718. doi: 10.1136/bmjgh-2019-001718 (PMC7042608; doi:10.1136/bmjgh-2019-001718)
Supplement: Supplementary data [file bmjgh-2019-001718supp004.pdf]

Supplementary Table 3: Effect of WASH and IYCF interventions on Early Child Development outcomes at 24 months in HIV-exposed uninfected and HIV-unexposed children

CONTINUOUS OUTCOMES	Treatment group	HIV-exposed uninfected		HIV-unexposed		Difference between means (95% CI)	*P*
		N	Mean score (SD)	N	Mean score (SD)		
Malawi Developmental Assessment (MDAT) Tool	SOC	63	90.7 (8.1)	373	92.7 (9.5)	-1.80 (-3.70, 0.10)	0.063
	IYCF	63	91.8 (8.6)	389	93.0 (8.6)	-1.02 (-3.01, 0.97)	0.315
	WASH	79	89.6 (9.2)	406	91.6 (9.2)	-1.90 (-3.79, -0.01)	0.048
	IYCF+WASH	95	95.5 (9.0)	433	93.8 (9.2)	1.50 (-0.05, 3.04)	0.057
MacArthur Bates CDI (Vocabulary)	SOC	63	56.9 (18.3)	365	61.3 (18.7)	-4.24 (-8.27, -0.22)	0.039
	IYCF	62	58.4 (20.7)	375	61.3 (18.6)	-2.73 (-8.60, 3.14)	0.362
	WASH	75	58.4 (18.9)	397	61.1 (19.1)	-2.70 (-7.28, 1.87)	0.247
	IYCF+WASH	91	66.0 (15.9)	423	63.3 (18.8)	2.55 (-0.91, 6.02)	0.148
A-not-B Test (object permanence)	SOC	52	7.8 (1.3)	351	7.8 (1.3)	0.02 (-0.40, 0.43)	0.943
	IYCF	59	7.6 (1.3)	364	7.7 (1.4)	-0.10 (-0.43, 0.23)	0.546
	WASH	72	7.8 (1.5)	372	7.7 (1.4)	0.05 (-0.30, 0.39)	0.784
	IYCF+WASH	86	7.7 (1.3)	404	7.9 (1.4)	-0.14 (-0.52, 0.23)	0.459
**DICHOTOMOUS OUTCOMES**	**Treatment group**	**N**	**n (%)**	**N**	**n (%)**	**Unadjusted Relative Risk (95% CI)**	***P***
Self-control Task (Hidden)	SOC	59	43 (72.9%)	365	237 (64.9%)	1.13 (0.96, 1.34)	0.129
	IYCF	63	39 (61.9%)	383	245 (64.0%)	0.96 (0.78, 1.20)	0.744
	WASH	78	47 (60.3%)	396	252 (63.6%)	0.94 (0.77, 1.16)	0.573
	IYCF+WASH	94	65 (69.2%)	432	283 (65.5%)	1.06 (0.93, 1.20)	0.415
Self-control Task (Unhidden)	SOC	58	31 (53.5%)	359	170 (47.4%)	1.13 (0.87, 1.47)	0.346
	IYCF	63	29 (46.0%)	381	172 (45.2%)	1.02 (0.77, 1.34)	0.895
	WASH	77	30 (39.0%)	394	173 (43.9%)	0.89 (0.68, 1.16)	0.383
	IYCF+WASH	92	43 (46.7%)	429	201 (46.9%)	1.00 (0.81, 1.23)	0.998
